# Continuation Versus Discontinuation of Nonselective Beta-Blockers After Transjugular Intrahepatic Portosystemic Shunt Placement: A Real-World, Target-Trial Emulation Analysis

**DOI:** 10.3390/jcm15155881

**Published:** 2026-07-28

**Authors:** Ali Emre Bardak, Ayse Ipek Bardak, Gizem Teker, Hind El Naamani, Elif Gokcek Uskudar, Volkan Senkal, Bilger Cavus, Nazli Begum Ozturk, Ahmet Gurakar

**Affiliations:** 1 Department of Medicine, Boston Medical Center-Brighton, Boston, MA 02135, USA; aliemre.bardak@bmc.org (A.E.B.); hind.elnaamani@bmc.org (H.E.N.); 2 Center for Computational Molecular Biology, Brown University, Providence, RI 02906, USA; ayse_ipek_bardak@brown.edu; 3 Beth Israel Deaconess Medical Center, Boston, MA 02215, USA; 4 Division of Gastroenterology and Hepatology, Johns Hopkins University School of Medicine, Baltimore, MD 21218, USA; 5 Division of Gastroenterology and Hepatology, Istanbul University, Istanbul 34452, Türkiye; 6 Division of Gastroenterology, Saint Louis University School of Medicine, St. Louis, MO 63104, USA; begum.ozturk@ssmhealth.com

**Keywords:** cirrhosis, hepatic encephalopathy, nonselective beta-blockers, portal hypertension, transjugular intrahepatic portosystemic shunt

## Abstract

**Background/Objectives**: Nonselective beta-blockers (NSBBs) are foundational for variceal bleeding prophylaxis in cirrhosis. After transjugular intrahepatic portosystemic shunt (TIPS) placement, portal pressure is mechanically decompressed, and practice guidance generally supports discontinuing NSBBs when shunt function is adequate and no other indication exists; however, real-world adoption and outcomes associated with post-TIPS NSBB strategies remain incompletely characterized in large, multicenter populations. **Methods**: We performed a real-world, target-trial emulation in the TriNetX U.S. Collaborative Network using a prespecified 90-day landmark. Adults (≥18 years) with cirrhosis undergoing first TIPS who had NSBB prescribed in the prior year and who survived to post-TIPS day 90 were included. Strategy was classified during days 0–90 (continuation: ≥1 NSBB prescription; discontinuation: none). Propensity score matching (1:1) balanced demographics, comorbidities, portal hypertension complications, and laboratory values (including the components of the Freiburg Index of Post-TIPS Survival [FIPS], Model for End-stage Liver Disease–Sodium [MELD-Na], and Child–Pugh scores). Follow-up began at day 90 and continued through day 365. Primary outcomes were overall survival and transplant-free survival; secondary outcomes were hepatic encephalopathy (HE), esophageal variceal bleeding (EVB), and ICU admission. **Results**: Among 5111 patients (continuation: *n* = 2558; discontinuation: *n* = 2553), 2180 matched pairs were analyzed. One-year survival was similar (89.3% vs. 89.6%; HR: 1.03; 95% CI: 0.84–1.27), as was transplant-free survival (78.8% vs. 77.9%; HR: 0.95; 95% CI: 0.82–1.10). The cause-specific hazard of HE was higher with continuation (HR; 1.29; 95% CI: 1.14–1.46), while those of EVB (HR: 1.08; 95% CI: 0.90–1.29) and ICU admission (HR: 1.02; 95% CI: 0.86–1.22) were similar. Results were consistent in both strict discontinuation and adherence-based sensitivity analyses. **Conclusions**: In stabilized post-TIPS patients with prior NSBB use, continuation was not associated with improved 1-year survival or transplant-free survival and was associated with a higher cause-specific hazard of HE.

## 1. Introduction

Nonselective beta-blockers (NSBBs) are foundational for primary and secondary prophylaxis of variceal hemorrhage in cirrhosis [1]. After transjugular intrahepatic portosystemic shunt (TIPS) placement, portal pressure is mechanically decompressed, and the portal hypertension-driven indication for NSBB therapy may become unnecessary when adequate shunt function is achieved [2,3]. Consistent with this physiologic rationale, contemporary practice guidance supports the discontinuation of NSBBs after effective TIPS in the absence of other indications for beta-blockade, while selective continuation of therapy is appropriate when there are non–portal hypertension indications or when portal decompression is inadequate [4,5,6].

Although guidance provides a management framework, real-world implementation may vary across health systems and clinical contexts, and contemporary multicenter data linking post-TIPS NSBB strategies to downstream outcomes remain relatively limited [3]. Patients undergoing TIPS often have advanced decompensation and hemodynamic vulnerability, and the balance of potential benefits versus harms of continued beta-blockade in this setting has not been well characterized across broad populations [5,6,7,8].

To complement existing evidence and better contextualize guideline-consistent care in contemporary practice, we conducted a large, real-world comparative effectiveness study using the TriNetX U.S. Collaborative Network and an emulated target-trial framework with a 90-day landmark. Among adults with cirrhosis who underwent TIPS, had evidence of NSBB prescription in the year prior to the procedure, and survived to post-TIPS day 90, we compared outcomes associated with NSBB continuation versus discontinuation strategies.

## 2. Materials and Methods

### 2.1. Study Design and Data Source

We conducted a retrospective, real-world, comparative cohort study using the TriNetX U.S. Collaborative Network (TriNetX, LLC, Cambridge, MA, USA), a federated research platform aggregating de-identified electronic health record (EHR) data from participating health systems across the United States. The data available in TriNetX include demographics, diagnoses, procedures, laboratory measurements, medications, clinical outcomes, and encounter-level utilization. Diagnoses and clinical events were identified using ICD-10-CM codes, procedures were identified using CPT and/or ICD procedure codes, and medication prescription was identified using standardized medication concepts within TriNetX. All data were de-identified in accordance with the Health Insurance Portability and Accountability Act (HIPAA), and analyses conducted using the TriNetX research network, which contains only de-identified data, are considered institutional review board-exempt by Boston University.

### 2.2. Target-Trial Emulation Framework and Landmark Design

We designed the analysis using a target-trial emulation framework (Table 1). We prespecified eligibility criteria, a strategy assignment window, time zero, follow-up, outcomes, estimands, and analytic methods to strengthen temporality and reduce bias. Specifically, we emulated a pragmatic trial comparing two post-TIPS management strategies for NSBBs among adults with cirrhosis who underwent TIPS, had evidence of NSBB prescription within the year prior to the procedure, and survived to post-TIPS day 90. The strategy assignment was defined during the first 90 days after TIPS, and outcome follow-up began at the 90-day landmark and continued through 1 year after TIPS. This landmark design was implemented to focus on a stabilized post-procedural population and to separate strategy classification from subsequent outcome ascertainment.

The primary estimand was the intention to treat (ITT)-like effect of early post-TIPS NSBB continuation versus discontinuation on 1-year overall survival and transplant-free survival, with secondary estimands evaluating cirrhosis-related complications.

### 2.3. Cohort Identification and Eligibility Criteria

Adults aged ≥ 18 years with cirrhosis who underwent their first TIPS were identified using diagnosis and procedure codes. To restrict the cohort to patients in whom post-TIPS NSBB management was clinically relevant, we required evidence of NSBB prescription within the year before TIPS (propranolol, nadolol, or carvedilol). The TIPS procedure date served as the index date. Patients who did not survive to post-TIPS day 90 were excluded.

Post-TIPS NSBB strategy was classified during days 0–90 after TIPS. Patients were categorized as continuing NSBB therapy if at least one NSBB prescription was recorded during days 0–90. For the primary landmark analysis, patients were categorized as discontinuing NSBB therapy if no NSBB prescriptions were recorded during days 0–90. Patients were analyzed according to their initial strategy classification and were not reclassified after follow-up began on day 90.

Detailed code definitions for cohort construction, exposure classification, and outcome ascertainment (ICD-10-CM, CPT, and RxNorm) are provided in the Supplementary Materials.

### 2.4. Outcomes

Outcome follow-up began at post-TIPS day 90 and continued through post-TIPS day 365, death, or the end of available observation (whichever occurred first). The primary outcomes were overall survival and transplant-free survival. Overall survival was defined as the time from the 90-day landmark to all-cause death. Transplant-free survival was defined as the time from the 90-day landmark to liver transplantation or death (whichever occurred first).

Secondary outcomes included hepatic encephalopathy (HE), esophageal variceal bleeding (EVB), and intensive care unit (ICU) admission and were assessed from the landmark through one year using diagnosis codes, procedure codes, and encounter constructs available in TriNetX (Supplementary Materials).

### 2.5. Propensity Score Matching

To minimize confounding, patients continuing NSBB therapy were matched 1:1 to those discontinuing therapy by nearest-neighbor matching without replacement, with propensity scores estimated by logistic regression within TriNetX.

Covariates were prespecified and ascertained in the 12 months preceding TIPS, including demographics (age, sex, and race), body mass index (BMI), liver disease etiology, cirrhosis- and portal hypertension-related complications (ascites, HE, EVB, spontaneous bacterial peritonitis, and hepatorenal syndrome), comorbidities (hypertension, diabetes mellitus, chronic kidney disease, heart failure, and ICU admission in the preceding year), and laboratory values (hemoglobin, platelet count, sodium, creatinine, albumin, total bilirubin, and international normalized ratio [INR]), which, together with age, encompass the components of the Model for End-Stage Liver Disease–Sodium (MELD-Na), Child–Pugh, and Freiburg Index of Post-TIPS Survival (FIPS) scores. The FIPS was derived from pre-procedure variables specifically to predict post-TIPS survival [9] and also predicts post-TIPS HE, which is a secondary outcome of this study [10]. All matching covariates were measured before the shared TIPS procedure. The day-90 variables were not used for matching, as they may be consequences of the assigned strategy or may have happened before the strategy-determining event occurred, introducing bias. Covariate balance before and after matching was assessed using standardized mean differences (SMDs). An SMD <0.20 was prespecified as indicating adequate balance, and balance was additionally evaluated against the more conservative <0.10 threshold [11].

### 2.6. Statistical Analysis

Baseline characteristics were summarized with descriptive statistics. Time-to-event outcomes were analyzed by the Kaplan–Meier method and compared with the log-rank test, and Cox proportional-hazards models were used to estimate hazard ratios (HRs) with 95% confidence intervals (CIs). Patients were censored at the date of their last recorded EHR activity, so loss to follow-up or transfer outside the network was treated as right-censoring. For each outcome, we also report the risk ratio (RR), the risk difference (RD), and the Kaplan–Meier survival probability, each with 95% CIs. The RR and RD were computed as crude cumulative risks by day 365 (the proportion of patients in each group with the event by 1 year) and are not adjusted for censoring. Therefore, they can differ from the difference in Kaplan–Meier probabilities, which accounts for censoring and differential follow-up between groups. We prespecified the HR as the primary effect measure because it is the conventional summary for time-to-event outcomes. We acknowledge that the HR is non-collapsible and has known limitations as a stand-alone causal estimand [12]; thus, we report the collapsible RR and RD alongside every HR. The proportional-hazards assumption was tested for every outcome using the proportionality test provided by the TriNetX platform. For the non-fatal secondary outcomes, for which death is a competing event (hepatic encephalopathy, esophageal variceal bleeding, and ICU admission), the prespecified primary estimand was the cause-specific hazard ratio, i.e., the effect of the NSBB strategy on the instantaneous rate of the event among patients still alive and event-free. On the absolute-risk scale, the estimand of interest is the total effect on the cumulative incidence of the event, which, under competing mortality, is most appropriately estimated with a cause-specific cumulative-incidence (Aalen–Johansen) or subdistribution (Fine–Gray) approach [13]. Because neither estimator was available within the federated TriNetX platform, we report the cause-specific hazard ratio as the primary measure and present the Kaplan–Meier event-free probabilities descriptively, recognizing that, by treating death as censoring, they can overestimate the absolute cumulative incidence of these events when the competing risk of death is non-negligible [14]. Analyses used the built-in analytic tools of TriNetX. All analyses were performed on 18 July 2026. All tests were two-sided, and *p* < 0.05 was considered statistically significant.

### 2.7. Crossover Assessment After the Landmark

To contextualize the implementation of the prespecified post-TIPS NSBB strategies and to assess the ITT-like landmark estimands, we performed a descriptive analysis of NSBB prescription orders recorded during follow-up (post-TIPS days 90–365) in each group. This assessment was primarily intended to evaluate potential crossover/re-initiation among patients classified as discontinuers. Because TriNetX medication data reflect EHR prescription orders and do not reliably capture pharmacy fills, adherence, dose changes, or days’ supply/refill duration, this analysis was intended as a qualitative crossover check rather than a measure of medication adherence or persistence.

### 2.8. Sensitivity Analysis

The additional analyses served three complementary purposes, varying the exposure definition, restricting to incident events, and corroborating the coded HE outcome with an independent medication proxy. To evaluate the robustness of the primary findings to exposure misclassification and post-landmark crossover, we performed two sensitivity analyses. First, a strict-discontinuation comparator required no recorded NSBB prescription order at any time after TIPS through day 365, while the continuation strategy remained defined by at least one NSBB order during days 0–90. Second, an adherence-based (per-protocol-like) analysis compared sustained NSBB exposure, defined by an NSBB prescription also recorded during months 3–12, with no post-TIPS NSBB. In both analyses, propensity score matching, the 90-day landmark, the follow-up window (days 90–365), and the outcome definitions were identical to the primary analysis. Because both exposure definitions incorporate information recorded after the landmark and the adherence-based analysis is additionally subject to immortal time bias due to sustained exposure requiring a prescription recorded after the landmark, they are interpreted as supportive and directional rather than as primary estimates [15]. A formal per-protocol estimator using cloning with inverse probability-of-censoring weighting was not feasible because the federated, de-identified platform does not expose patient-level, time-varying data. We further repeated the HE and EVB analyses among patients without a recorded diagnosis of the respective event before the follow-up window to distinguish incident from recurrent events. As a corroborating analysis, we compared the incidence of new HE-directed pharmacotherapy (lactulose, rifaximin, and either agent) between strategies as an independent proxy for the coded HE outcome.

### 2.9. Subgroup Analyses

Subgroup analyses were prespecified and conducted by age, sex, liver disease etiology, and selected baseline clinical vulnerability markers (i.e., heart failure, prior HE, and prior EVB). Subgroup findings were interpreted as exploratory.

### 2.10. Declaration of AI Use

During the preparation of this manuscript, the authors used Claude Opus 4.8 (Anthropic, San Francisco, CA, USA) for the purposes of language editing. The authors have reviewed and edited the output and take full responsibility for the content of this publication.

## 3. Results

### 3.1. Study Population and Baseline Characteristics

**Figure 1:**
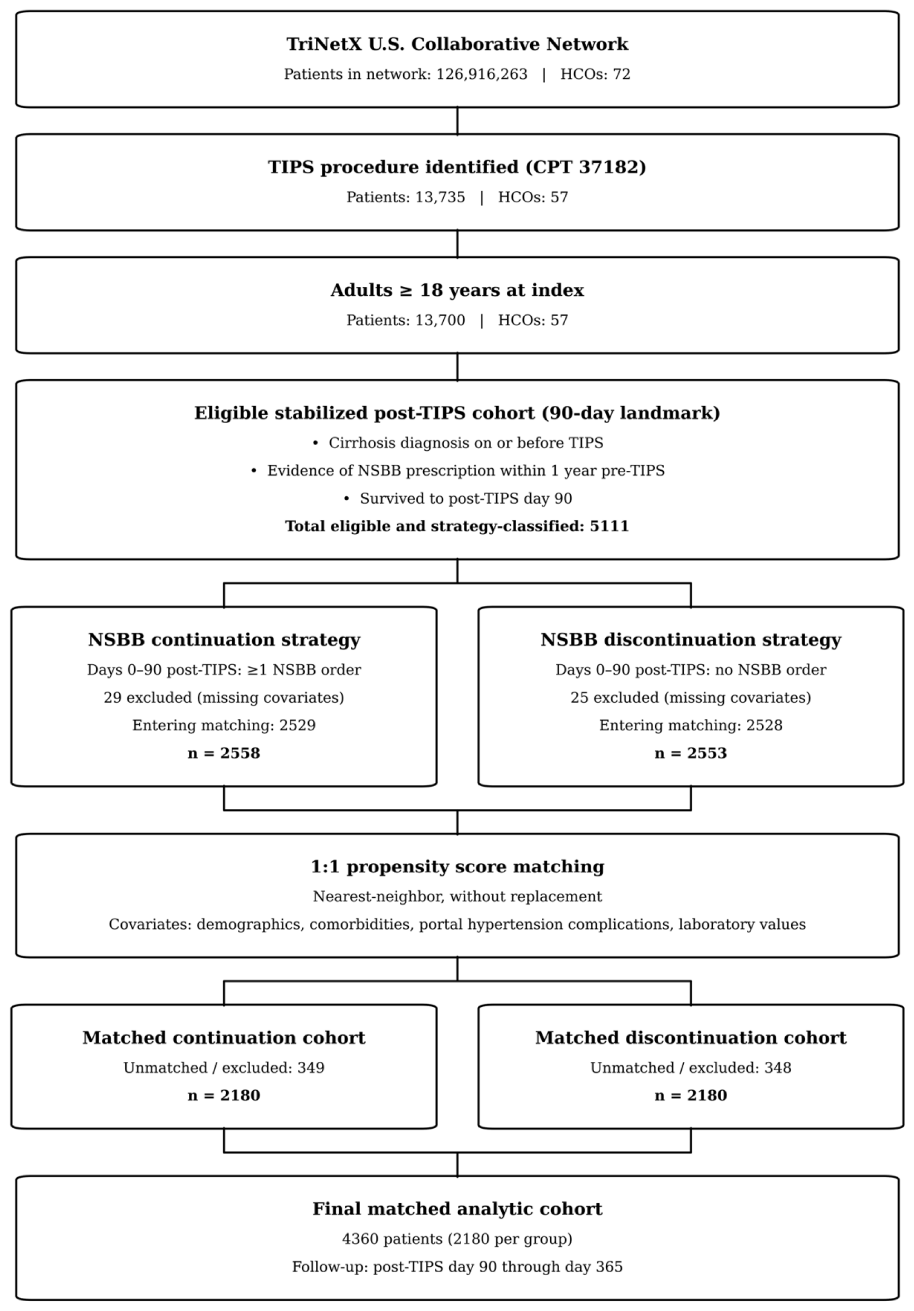
Cohort selection and propensity score-matched analytic sample. Adults (≥18 years) undergoing TIPS (CPT 37182) in the TriNetX U.S. Collaborative Network with cirrhosis, NSBB use within 1 year pre-TIPS, and survival to post-TIPS day 90 were classified during days 0–90 as continuation (≥1 prescription; *n* = 2558) or discontinuation (none; *n* = 2553). After excluding patients with missing covariates, 1:1 propensity score matching yielded 2180 patients per group (final *n* = 4360). Follow-up extended from day 90 to day 365. Abbreviations: TIPS, transjugular intrahepatic portosystemic shunt; NSBB, nonselective beta-blocker; HCO, healthcare organization.

At the time of analysis, the TriNetX U.S. Collaborative Network comprised 126,916,263 patients across 72 healthcare organizations. Among adults with cirrhosis who had evidence of NSBB use in the year before TIPS and who survived to post-TIPS day 90, 5111 were classified by post-TIPS NSBB strategy (2558 continuation and 2553 discontinuation). After 1:1 propensity score matching, 2180 patients remained in each group (Figure 1). The median follow-up was 365 days in both groups, and the mean follow-up was similar between the two strategies (295 vs. 276 days).

In the matched cohort, the mean age was 56.9 years; 66% of patients were male, and 82% were White; and alcohol-related cirrhosis was the most common etiology (53%). After matching, demographics, comorbidities, cirrhosis- and portal hypertension-related complications, and laboratory parameters were balanced between the two cohorts (Table 2). All SMDs were below the prespecified 0.20 threshold, and most were below 0.10. The largest between-group difference was in serum sodium (136.5 vs. 135.8 mEq/L; SMD 0.16) for the continuation and discontinuation groups, respectively. The remaining covariates with an SMD above 0.10 are marked in Table 2. The components of the MELD-Na, Child–Pugh, and FIPS scores were balanced after matching.

### 3.2. Primary Clinical Outcomes at One Year

**Figure 2:**
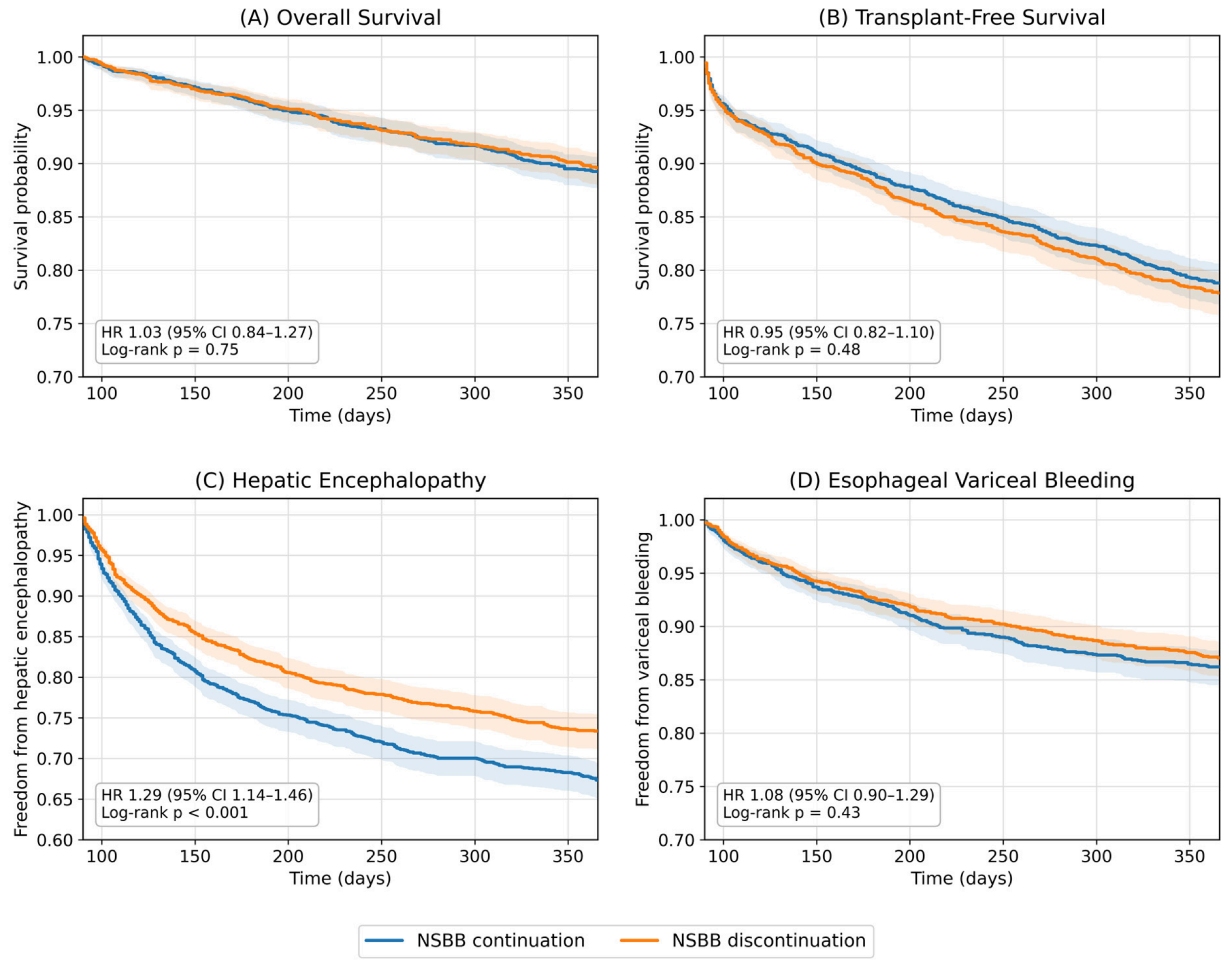
Kaplan–Meier curves for one-year outcomes by post-TIPS NSBB strategy in the 1:1 propensity score-matched cohort (2180 per group), from the 90-day landmark through day 365. (**A**) Overall survival (time to all-cause death). (**B**) Transplant-free survival (time to liver transplantation or death). (**C**) Freedom from hepatic encephalopathy. (**D**) Freedom from esophageal variceal bleeding. Blue, NSBB continuation; orange, NSBB discontinuation; shaded bands, 95% CIs. Each panel is annotated with the hazard ratio (95% CI) for continuation versus discontinuation and the log-rank *p*-value; the proportional-hazards assumption was satisfied for every outcome (proportionality-test p: overall survival, 0.98; transplant-free survival, 0.53; hepatic encephalopathy, 0.36; ICU, 0.70; esophageal variceal bleeding, 0.40). For the non-fatal outcomes (**C**,**D**), curves are Kaplan–Meier estimates that treat death as a censoring event and therefore represent cause-specific, death-censored event-free probabilities that can overstate absolute cumulative incidence when death competes. The cause-specific hazard ratio is the primary measure for these outcomes. The federated TriNetX platform does not export the time-varying risk set or patient-level censoring times, so a numbers-at-risk table and individual censoring marks are not available. CI, confidence interval; NSBB, nonselective beta-blocker; TIPS, transjugular intrahepatic portosystemic shunt.

In the propensity score-matched cohort (2180 per group), followed from the prespecified 90-day landmark through 1 year post TIPS, overall survival was similar between the NSBB continuation and discontinuation strategies (Kaplan–Meier one-year survival: 89.3% vs. 89.6%; HR: 1.03; 95% CI: 0.84–1.27; log-rank *p* = 0.75; Figure 2A). Transplant-free survival was likewise comparable (78.8% vs. 77.9%; HR: 0.95; 95% CI: 0.82–1.10; log-rank *p* = 0.48; Figure 2B). The proportional-hazards assumption held for both outcomes (proportionality-test *p* > 0.05; Table 3), so each HR summarizes an approximately constant relative effect over the one-year follow-up. Event counts, crude proportions, and risk-scale estimates for all outcomes are provided in Table 3. Because follow-up was near-complete through one year, the Kaplan–Meier overall-survival estimates were close to the corresponding crude proportions.

### 3.3. Secondary Clinical Outcomes at One Year

The cause-specific hazard of HE was higher with NSBB continuation than discontinuation (HR: 1.29; 95% CI; 1.14–1.46; log-rank *p* < 0.01; Figure 2C). In an additional analysis, continuation was also associated with a higher incidence of HE-directed pharmacotherapy, including treatment with rifaximin (HR: 1.18; 95% CI: 1.06–1.31; log-rank *p* < 0.01), lactulose (HR: 1.17; 95% CI: 1.06–1.29; log-rank *p* < 0.01), and either agent (HR: 1.15; 95% CI: 1.06–1.26; log-rank *p* < 0.01). By contrast, ICU admission (HR: 1.02; 95% CI: 0.86–1.22; log-rank *p* = 0.82) and EVB (HR: 1.08; 95% CI: 0.90–1.29; log-rank *p* = 0.43; Figure 2D) did not differ between strategies. The proportional-hazards assumption held for all three outcomes (proportionality-test *p* > 0.05). Crude proportions and risk-scale estimates for all outcomes are summarized in Table 3.

### 3.4. Crossover After the Landmark

As a descriptive strategy check, we evaluated NSBB prescription documentation during follow-up (post-TIPS days 90–365). In the continuation cohort, 731 of 2180 (33.5%) patients had ≥1 additional NSBB prescription order recorded after the landmark. In the discontinuation cohort, 248 of 2180 (11.4%) patients had ≥1 NSBB prescription order recorded during follow-up. Overall, the distribution of recorded prescription-order counts differed significantly between strategies (*p* < 0.0001).

Because medication data reflect EHR prescription documentation rather than pharmacy fill records and a single prescription may include extended refills or a longer intended duration, these counts should be interpreted as documentation of prescription rather than a quantitative measure of treatment persistence. Consistent with the landmark ITT-like framework, patients were analyzed according to their days 0–90 strategy classification and were not reclassified based on post-landmark prescriptions.

### 3.5. Sensitivity Analyses

We performed two sensitivity analyses to address exposure misclassification and post-landmark crossover. Full results are provided in Table S1.

In the strict-discontinuation comparator, in which the discontinuation group had no recorded NSBB order at any time after TIPS (1859 matched pairs), neither overall survival (HR: 0.89; 95% CI: 0.72–1.11; log-rank *p* = 0.29) nor transplant-free survival (HR: 0.90; 95% CI: 0.77–1.05; log-rank *p* = 0.18) differed between continuation and strict discontinuation, and HE remained more frequent with continuation (HR: 1.40; 95% CI: 1.22–1.61; log-rank *p* < 0.001), whereas ICU admission (HR: 1.13; 95% CI: 0.93–1.38; log-rank *p* = 0.22) and EVB (HR: 1.10; 95% CI; 0.89–1.36; log-rank *p* = 0.38) did not differ. Because the strict-discontinuation comparator is defined by the absence of any NSBB order through day 365, it conditions on post-landmark information and can bias the survival estimates in favor of continuation. These results are therefore interpreted as directional.

In the adherence-based (per-protocol-like) analysis comparing sustained NSBB exposure with no post-TIPS NSBB (828 matched pairs), overall survival (HR: 0.85; 95% CI; 0.63–1.15; log-rank *p* = 0.29) and transplant-free survival (HR: 0.94; 95% CI: 0.76–1.16; log-rank *p* = 0.57), again, did not differ, whereas HE (HR: 1.73; 95% CI: 1.44–2.07; *p* < 0.001), ICU admission (HR: 1.39; 95% CI: 1.08–1.79; log-rank *p* = 0.01), and EVB (HR: 1.71; 95% CI: 1.28–2.29; log-rank *p* < 0.001) were all more frequent with sustained exposure. Because sustained exposure requires a prescription recorded after the landmark, the sustained-exposure group was followed substantially longer (mean 345 vs. 258 days), introducing immortal time bias that both inflates cumulative complication rates and can produce a survival advantage in the longer-followed continuation group. In a further analysis within the same 2180 matched pairs, restricting the outcome to patients free of the respective event before the follow-up window, continuation remained associated with a higher cause-specific hazard of new HE at a magnitude consistent with the primary analysis (HR: 1.21; 95% CI: 0.93–1.57; log-rank *p* = 0.15). The wider confidence interval reflects the smaller at-risk set rather than attenuation of the effect. Among the matched patients free of EVB before the follow-up window, incident EVB, again, did not differ between strategies, as in the primary analysis (HR: 1.04; 95% CI: 0.58–1.86; log-rank *p* = 0.90).

### 3.6. Subgroup Analyses

Exploratory subgroup analyses are summarized in the forest plots (Supplementary Materials). The associations between NSBB continuation and both overall survival and transplant-free survival were generally consistent across prespecified demographic, etiologic, and clinical vulnerability subgroups; the only exception was a nominally lower mortality hazard with continuation in the youngest patients (age 18–59), which was not accompanied by a corresponding difference in transplant-free survival. For HE, the increased hazard associated with NSBB continuation was directionally consistent across all strata and statistically significant in most, and it appeared more pronounced in older patients (≥60) than in younger patients (<60). Subgroup findings for EVB and ICU admission were broadly concordant with the primary analyses, with no subgroup showing a benefit for continuation, and should be interpreted cautiously, given the exploratory nature of subgroup comparisons.

## 4. Discussion

In this large, multicenter, real-world target-trial emulation of patients with cirrhosis who survived the early post-procedural period after TIPS, we found that continuing NSBB therapy was not associated with improved 1-year overall survival or transplant-free survival compared with discontinuation. In contrast, continuation of NSBB therapy was associated with a higher cause-specific hazard of HE, while rates of EVB and ICU admission were similar between groups. These findings were generally consistent across prespecified subgroups and were further supported by sensitivity analyses, including strict-discontinuation and adherence-based comparators, which yielded concordant results.

The role of NSBB therapy in clinically significant portal hypertension (CSPH) is well established, including with respect to the prevention of decompensation and variceal bleeding [4]. However, once a TIPS is placed and portal pressure is mechanically decompressed, often below the threshold defining CSPH, the incremental hemodynamic benefit of continued beta-blockade becomes less certain [5]. While there is no major controversy regarding the physiologic effects of NSBBs, post-TIPS management remains heterogeneous in clinical practice, and prospective randomized data are limited. In this context, our findings contribute contemporary, large-scale comparative effectiveness data regarding strategy-level decisions after TIPS.

Importantly, our study was designed to focus on stabilized patients by implementing a prespecified 90-day landmark. Patients who did not survive to post-TIPS day 90 were excluded, and the treatment strategy was classified during the first 90 days, with follow-up beginning thereafter. This design reduces immortal time bias and minimizes confounding from acute post-procedural instability, including early hemodynamic shifts, bleeding, or peri-interventional complications. By isolating a clinically stable population, our analysis specifically evaluates the association between ongoing NSBB management and medium-term outcomes rather than acute peri-procedural events.

Our findings align with recent observational data suggesting limited benefit of NSBB continuation after TIPS. In one of the largest dedicated post-TIPS NSBB cohorts to date, Tiede et al. evaluated 305 patients and found that NSBB use at TIPS placement and continuation after discharge were not associated with lower risks of mortality or hepatic decompensation in adjusted competing-risk analyses; in a prospective subset (*n* = 45), NSBB exposure was not linked to lower levels or more favorable trajectories of WBC, CRP, IL-6, or any of the analyzed inflammatory markers [3]. While NSBBs are highly effective for primary and secondary prophylaxis of variceal bleeding in non-TIPS populations, mechanical portal decompression via TIPS appears to mitigate the need for additional pharmacologic portal-pressure reduction in most patients [16]. In our matched cohort, continuation of NSBB therapy did not reduce variceal bleeding rates, supporting the concept that once portal pressure is adequately controlled by TIPS, the marginal benefit of NSBBs for bleeding prevention may be attenuated.

The increased cause-specific hazard of HE observed among patients who continued NSBB therapy after TIPS is clinically relevant and warrants careful physiologic consideration. Post-TIPS HE is primarily driven by an abrupt augmentation of portosystemic shunting, which diverts ammonia-rich portal blood directly into the systemic circulation, bypassing first-pass hepatic detoxification and increasing cerebral exposure to neurotoxins [5,17]. Overt HE occurs in approximately 30–50% of patients following TIPS, with severe or recurrent episodes in a substantial minority [18,19,20]. The pathophysiologic background is therefore already established at the time of shunt creation. In this altered hemodynamic state, the continuation of NSBB therapy may further modify systemic and organ-level perfusion. NSBBs reduce cardiac output through β_1_-adrenergic blockade and blunt compensatory increases in heart rate and contractility. In compensated cirrhosis, this reduction in cardiac output lowers portal inflow and is therapeutically advantageous [4]. However, in decompensated cirrhosis (particularly after TIPS insertion, when cardiac preload acutely increases), adequate cardiac reserve is required to maintain systemic perfusion [21]. This reserve is further constrained in advanced disease, in which the cardiac response to beta-blockade is, itself, blunted, the fall in cardiac index being roughly half that seen in compensated cirrhosis [22]. TIPS increases venous return and may unmask subclinical cirrhotic cardiomyopathy; impaired contractile response in this setting has been associated with adverse outcomes [7]. Continued β-blockade may limit the ability to adapt to this preload shift, potentially resulting in a lower effective arterial blood volume and reduced hepatic and renal arterial perfusion [7,23]. This hemodynamic framework provides a plausible link between NSBB continuation and HE. Reduced hepatic arterial flow may further impair ammonia metabolism in a liver already bypassed by the portosystemic shunt, while diminished renal perfusion may decrease renal ammonia excretion, compounding systemic hyperammonemia [24,25]. Observational data in non-TIPS populations showed an independent association between NSBB use and both covert and overt HE in patients with decompensated cirrhosis, supporting the concept that in advanced circulatory dysfunction, β-blockade may contribute to neurocognitive vulnerability [25]. Within the conceptual framework of the therapeutic-window hypothesis, the hemodynamic benefits of NSBBs diminish as circulatory reserve narrows and may become counterproductive in advanced disease [8,26]. After effective portal decompression by TIPS, the portal pressure-lowering effects of NSBBs become largely redundant, whereas their systemic hemodynamic effects persist [3]. In this context, continuation of NSBB therapy could plausibly contribute to HE in patients with limited cardiac and circulatory reserve.

Because this is an observational emulation, the causal interpretation of our estimates is bounded by potential residual confounding from unmeasured post-TIPS factors; the target-trial framework aligns eligibility, time zero, and the estimand but does not, by itself, guarantee causal identification. Nonetheless, the consistency of the HE signal across subgroups, sensitivity analyses, the concordant increase in HE-directed pharmacotherapy, and the directionally consistent event-naive (incident HE) analysis, combined with contemporary data demonstrating no survival or decompensation benefit from continuing NSBB after TIPS, suggests that routine continuation of NSBB therapy in stabilized post-TIPS patients may not provide net benefit and could increase HE susceptibility in selected patients. The most plausible direction of confounding by indication, discontinuation of NSBBs in more hemodynamically fragile patients, would bias toward worse outcomes among discontinuers, rendering the observed excess of HE with continuation likely conservative. A parallel argument applies to competing risk, since death occurred at a marginally higher crude frequency with continuation (8.6% vs. 7.8%) and, by removing patients from the HE risk set, would tend to suppress rather than inflate the observed HE excess. Notably, evidence on the prognostic weight of post-TIPS HE is mixed. Episodic overt HE was not associated with increased mortality in one cohort [27], whereas in a recent large cohort, it was found that overt HE raises long-term but not short-term mortality [28]. This is consistent with our findings, in which continuation was associated with more HE but no difference in one-year survival, suggesting that the clinical cost of this additional HE may emerge beyond the one-year horizon we examined rather than being absent, underscoring HE as a meaningful outcome, even when short-term survival is unchanged.

Beyond hemodynamics, NSBBs have been hypothesized to confer non-pressure-mediated benefits through modulation of systemic inflammation and bacterial translocation in CSPH, as suggested by trials demonstrating reduced decompensation with NSBB therapy in non-TIPS populations [4,29]. However, in the post-TIPS setting, a comprehensive analysis of biomarkers and clinical outcomes did not show attenuation of systemic inflammation or reduction in decompensation with continued NSBB therapy, suggesting that any anti-inflammatory signal observed before shunt creation may not translate once portal decompression has been achieved [3].

Our study was not designed to compare individual NSBB agents. Carvedilol has demonstrated superior portal-pressure reduction compared with traditional beta-blockers in non-TIPS settings and has shown favorable outcomes in certain secondary prophylaxis cohorts [16,30]. However, after effective TIPS placement, the relative portal hypotensive differences between agents may be less clinically relevant. Our primary objective was to evaluate post-TIPS management strategies rather than agent-level comparative effectiveness.

This study has several strengths. First, the target-trial emulation framework with a prespecified landmark strengthens temporal alignment between treatment strategy and outcome ascertainment. Second, comprehensive propensity score matching achieved balance across demographics, cirrhosis severity, portal hypertension-related complications, comorbidities, and laboratory parameters. Third, subgroup analyses demonstrated consistent findings across clinically relevant strata, enhancing internal validity. Fourth, the large multicenter dataset enhances generalizability to contemporary TIPS practice.

Several limitations merit consideration. First, this was a retrospective, observational analysis of de-identified EHR data; therefore, residual confounding cannot be fully excluded despite rigorous matching. In particular, confounding by indication may persist, as clinical factors influencing the decision to continue or discontinue NSBB (e.g., blood pressure, frailty, cardiac reserve, or perceived bleeding risk) are incompletely captured in structured EHR fields. In addition, matching covariates were ascertained at or before TIPS rather than at the 90-day landmark, so changes in clinical status occurring between TIPS and the day-90 treatment assignment that could influence both NSBB continuation and later outcomes were not balanced by the matching. Matching at the landmark would, in practice, have required a window around day 90 because few patients have data recorded on exactly that day, and such a window would, itself, introduce measurement error and a selection bias toward sicker patients who present to care more frequently. Restricting the incident analyses to event-free patients at the landmark and applying the strict-discontinuation comparator mitigate but do not eliminate this concern. The covariates matched at baseline are established pre-procedure determinants of post-TIPS prognosis, and because every patient in both arms underwent the same shunt procedure, a large differential change between strategies is unlikely. Moreover, the most plausible residual confounding, discontinuation of NSBB in the most hemodynamically fragile patients, would bias toward worse outcomes in the discontinuation arm and would therefore render the observed HE excess conservative. Second, we could not reliably distinguish whether pre-TIPS NSBB therapy was prescribed for primary or secondary prophylaxis of variceal bleeding, nor could we fully stratify by TIPS indication (e.g., refractory ascites versus variceal bleeding), urgency, procedural details, or center-level practice patterns, all of which may influence downstream outcomes. Third, medication strategies were defined using EHR prescription documentation and may not fully capture adherence, dose intensity, titration, pharmacy fills, days’ supply, or temporary treatment interruptions. Fourth, key physiologic and procedural variables, particularly post-TIPS portal-pressure gradients/shunt function, stent characteristics, and spontaneous portosystemic shunts, were unavailable, limiting assessment of whether incomplete portal decompression modified associations. Fifth, outcomes (including HE and EVB) were ascertained using diagnostic and procedural codes and may underestimate milder events or severity. Sixth, for nonfatal outcomes, death was treated as a censoring event within the platform’s survival framework; competing-risk methods were not available, and results should be interpreted as cause-specific hazards. Because the HR has recognized limitations as a stand-alone causal estimand [12], we additionally report the RR and RD for every outcome. The proportional-hazards assumption held for every outcome, so each HR reflects an approximately constant effect across the full one-year window rather than being confined to early follow-up. Seventh, the 90-day landmark design strengthens temporal validity but restricts inference to patients who survived and remained under observation through day 90, and follow-up was limited to 1 year, precluding conclusions about longer-term outcomes. Eighth, a formal per-protocol analysis using cloning with inverse probability-of-censoring weighting and subdistribution (Fine–Gray) competing-risk models, as well as the Aalen–Johansen estimator for the cumulative incidence of the non-fatal outcomes, could not be implemented within the aggregate TriNetX environment; we therefore report cause-specific hazards and adherence-based sensitivity analyses.

## 5. Conclusions

In this real-world cohort of patients surviving the early post-TIPS period, continuation of NSBB therapy was not associated with improved overall or transplant-free survival or with a reduction in variceal bleeding and was associated with a higher cause-specific hazard of HE at post-TIPS year 1. In stabilized patients with prior NSBB use, these findings support an individualized, guideline-consistent reassessment of NSBB therapy after effective portal decompression rather than routine continuation. Discontinuation in clinical practice must always be contingent on demonstration of an adequate shunt function and the absence of a persistent non-portal-hypertensive indication for beta-blockade. Prospective randomized data are needed to confirm these associations.

## Figures and Tables

**Table 1:** Target-trial emulation specification: NSBB continuation versus discontinuation after TIPS placement.

Protocol Component	Target Trial (Ideal Randomized Trial)	Emulation (This Study)
Eligibility	Adults with cirrhosis and a functioning TIPS who are receiving an NSBB and are candidates for either continuation or discontinuation.	Adults ≥ 18 years with cirrhosis undergoing first TIPS (CPT 37182), with an NSBB (propranolol, nadolol, or carvedilol) prescribed within a year before TIPS, and surviving to post-TIPS day 90.
Treatment strategies	(1) Continue NSBB therapy after TIPS; (2) discontinue NSBB therapy after TIPS.	Continuation: ≥1 NSBB prescription during post-TIPS days 0–90. Discontinuation: no NSBB prescription during days 0–90.
Assignment procedure	Random assignment to a strategy at time zero (pragmatic, unblinded).	Strategy classified for prescription from days 0 to 90; exchangeability approximated by 1:1 propensity score matching on prespecified pre-TIPS covariates.
Time zero	Start of follow-up at the 90-day landmark, when strategy is assigned.	Post-TIPS day 90 (landmark); survival to day 90 required.
Outcomes	Primary: overall survival and transplant-free survival. Secondary: hepatic encephalopathy, esophageal variceal bleeding, and ICU admission.	Same outcomes, ascertained using ICD-10-CM/CPT codes and encounter constructs within TriNetX.
Follow-up	From time zero to death, loss to follow-up, or 1 year.	From day 90 to death, last recorded EHR activity (right-censoring), or day 365.
Causal contrast/estimand	Intention-to-treat effect (primary) and per-protocol effect of the assigned strategy.	Intention-to-treat-like effect of the strategy assigned at the landmark; adherence-based (per protocol) contrasts reported as sensitivity analyses.
Statistical analysis	Kaplan–Meier and Cox models; risk ratios and risk differences; per-protocol analysis with appropriate adjustment for adherence.	Kaplan–Meier, log-rank, and Cox models within the matched cohort; risk ratios and risk differences; proportional-hazards assumption tested.
Subgroup and sensitivity analyses	Prespecified subgroups; robustness analyses.	Exploratory subgroups (age, sex, etiology, and vulnerability markers); strict-discontinuation and adherence-based sensitivity analyses.

Abbreviations: EHR, electronic health record; ICU, intensive care unit; NSBB, nonselective beta-blocker; TIPS, transjugular intrahepatic portosystemic shunt.

**Table 2:** Baseline characteristics before and after 1:1 propensity score matching by post-TIPS NSBB strategy. Continuous variables are expressed as mean ± SD, and categorical variables are expressed as *n* (%). SMDs are shown for each comparison. An SMD below 0.20 was prespecified as indicating adequate balance; after matching, covariates with an SMD between 0.10 and 0.20 are marked (§). AST, ALT, alkaline phosphatase, and direct bilirubin are shown for description only and were not included in the propensity score model.

Characteristic	Before PSM: Continuation	Before PSM: Discontinuation	SMD	After PSM: Continuation	After PSM: Discontinuation	SMD
Demographics						
Age at Index	57.1 ± 11.2	56.6 ± 11.2	0.04	56.9 ± 11.0	56.9 ± 11.1	0
Male	1689 (66.8%)	1615 (63.9%)	0.06	1438 (66.0%)	1440 (66.1%)	0
Female	839 (33.2%)	912 (36.1%)	0.06	741 (34.0%)	739 (33.9%)	0
White	2068 (81.8%)	2053 (81.2%)	0.01	1781 (81.7%)	1773 (81.3%)	0.01
Black or African American	146 (5.8%)	127 (5.0%)	0.03	120 (5.5%)	118 (5.4%)	0
Asian	51 (2.0%)	54 (2.1%)	0.01	46 (2.1%)	46 (2.1%)	0
BMI	29.4 ± 6.8	28.4 ± 6.3	0.15	29.2 ± 6.8	28.5 ± 6.3	0.11 §
Comorbidities						
Hypertension	1539 (60.9%)	1413 (55.9%)	0.10	1263 (57.9%)	1253 (57.5%)	0.01
Diabetes mellitus	1158 (45.8%)	999 (39.5%)	0.13	935 (42.9%)	929 (42.6%)	0.01
Chronic kidney disease	491 (19.4%)	545 (21.6%)	0.05	430 (19.7%)	415 (19.0%)	0.02
Heart failure	347 (13.7%)	281 (11.1%)	0.08	272 (12.5%)	255 (11.7%)	0.02
ICU admission in the preceding year	733 (29.0%)	837 (33.1%)	0.09	662 (30.4%)	667 (30.6%)	0
Liver disease *						
Alcoholic cirrhosis	1316 (52.0%)	1421 (56.2%)	0.08	1166 (53.5%)	1167 (53.5%)	0
Chronic HBV	60 (2.4%)	54 (2.1%)	0.02	46 (2.1%)	49 (2.2%)	0.01
Chronic HCV	477 (18.9%)	434 (17.2%)	0.04	394 (18.1%)	397 (18.2%)	0
MASH	645 (25.5%)	612 (24.2%)	0.03	561 (25.7%)	534 (24.5%)	0.03
Cirrhosis-related complications						
Ascites	1819 (71.9%)	1845 (73.0%)	0.02	1583 (72.6%)	1573 (72.2%)	0.01
Hepatic encephalopathy	619 (24.5%)	653 (25.8%)	0.03	533 (24.5%)	548 (25.1%)	0.02
Esophageal variceal bleeding †	724 (28.6%)	704 (27.8%)	0.02	596 (27.3%)	618 (28.3%)	0.02
Esophageal variceal bleeding ‡	941 (37.2%)	933 (36.9%)	0.01	786 (36.1%)	808 (37.1%)	0.02
Spontaneous bacterial peritonitis	228 (9.0%)	334 (13.2%)	0.13	225 (10.3%)	225 (10.3%)	0
Hepatorenal syndrome	158 (6.2%)	263 (10.4%)	0.15	158 (7.2%)	155 (7.1%)	0.01
Laboratory values (nearest to index)						
Hemoglobin (g/dL)	10.0 ± 2.2	9.7 ± 2.2	0.14	10.0 ± 2.2	9.7 ± 2.2	0.12 §
Platelet count (×10^3^/µL)	108.5 ± 78.8	108.1 ± 68.5	0.01	109.2 ± 80.0	107.7 ± 68.0	0.02
Sodium (mEq/L)	136.5 ± 4.2	135.7 ± 4.7	0.18	136.5 ± 4.3	135.8 ± 4.7	0.16 §
Creatinine (mg/dL)	1.10 ± 0.74	1.13 ± 0.80	0.05	1.09 ± 0.71	1.11 ± 0.76	0.02
Albumin (g/dL)	3.07 ± 0.65	3.08 ± 0.67	0	3.08 ± 0.64	3.08 ± 0.67	0
Bilirubin, total (mg/dL)	1.77 ± 2.57	2.36 ± 3.90	0.18	1.78 ± 2.48	2.22 ± 3.57	0.14 §
INR	1.37 ± 0.33	1.44 ± 0.40	0.17	1.38 ± 0.32	1.42 ± 0.36	0.12 §
Additional laboratory tests (not used for matching)						
AST (U/L)	50.7 ± 56.4	61.7 ± 313.2	0.05	51.1 ± 58.9	60.8 ± 323.1	0.04
ALT (U/L)	32.2 ± 43.4	34.7 ± 94.6	0.03	32.4 ± 45.7	34.8 ± 98.1	0.03
Alkaline phosphatase (U/L)	126.6 ± 87.0	129.7 ± 91.6	0.03	126.1 ± 86.5	131.2 ± 93.0	0.06
Bilirubin, direct (mg/dL)	0.81 ± 1.46	1.25 ± 2.67	0.20	0.83 ± 1.51	1.17 ± 2.52	0.16

* The etiological classifications were not mutually exclusive, as some patients had overlapping diagnoses. † ‡ Esophageal variceal bleeding was identified using ICD-10 codes I85.01 (†) and I85.11 (‡). § After matching, the SMD was between 0.10 and 0.20; these covariates met the prespecified balance criterion (SMD < 0.20) but not the more conservative 0.10 threshold. ALT, alanine aminotransferase; AST, aspartate aminotransferase; BMI, body mass index; HBV, hepatitis B virus; HCV, hepatitis C virus; INR, international normalized ratio; MASH, metabolic dysfunction-associated steatohepatitis; NSBB, nonselective beta-blocker; PSM, propensity score matching; SMD, standardized mean difference; TIPS, transjugular intrahepatic portosystemic shunt.

**Table 3:** One-year clinical outcomes by post-TIPS NSBB strategy in the 1:1 propensity score-matched cohort (2180 patients per group). Risk difference is in percentage points (continuation minus discontinuation); HRs and RRs are for continuation versus discontinuation.

Outcome	Continuation, *n* (%)	Discontinuation, *n* (%)	KM 1-yr, % (Cont. vs. Disc.)	HR (95% CI)	RR (95% CI)	Risk Difference, % (95% CI)	Log-Rank *p*
Overall survival (all-cause death)	188 (8.6)	170 (7.8)	89.3 vs. 89.6	1.03 (0.84–1.27)	1.11 (0.91–1.35)	+0.8 (−0.8 to +2.5)	0.75
Transplant-free survival (death or LT)	379 (17.4)	370 (17.0)	78.8 vs. 77.9	0.95 (0.82–1.10)	1.02 (0.90–1.17)	+0.4 (−1.8 to +2.7)	0.48
Hepatic encephalopathy	585 (26.8)	442 (20.3)	67.4 vs. 73.4	1.29 (1.14–1.46)	1.32 (1.19–1.47)	+6.6 (+4.0 to +9.1)	<0.001
Esophageal variceal bleeding	242 (11.1)	211 (9.7)	86.2 vs. 87.1	1.08 (0.90–1.29)	1.15 (0.96–1.37)	+1.4 (−0.4 to +3.2)	0.43
ICU admission	261 (12.0)	238 (10.9)	85.2 vs. 85.5	1.02 (0.86–1.22)	1.10 (0.93–1.29)	+1.1 (−0.8 to +2.9)	0.82

KM 1-year probability (continuation vs. discontinuation): survival for overall and transplant-free survival and freedom from the event for HE, ICU admission, and EVB. Proportional-hazards assumption satisfied for all outcomes (proportionality-test p: overall survival, 0.98; transplant-free survival, 0.53; HE, 0.36; ICU, 0.70; EVB, 0.40). For hepatic encephalopathy, ICU admission, and esophageal variceal bleeding, death is a competing event. The Kaplan–Meier estimates treat death as censoring and represent cause-specific event-free probabilities that can overstate absolute cumulative incidence, so the cause-specific HR is the primary measure for these outcomes. RR and RD are crude cumulative risks by day 365 (the proportion of patients with the event by 1 year), not adjusted for censoring or follow-up duration, and can therefore differ from the difference in Kaplan–Meier probabilities. CI, confidence interval; EVB, esophageal variceal bleeding; HE, hepatic encephalopathy; HR, hazard ratio; ICU, intensive care unit; KM, Kaplan–Meier; LT, liver transplantation; RD, risk difference; RR, risk ratio.

## Data Availability

The data supporting the findings of this study are available from the TriNetX Network. Restrictions apply to the availability of these data, which were used under license in the current study and are not publicly available. Data may be made available by the corresponding author upon reasonable request and with the permission of TriNetX.

## Supplementary Materials

The following supporting information can be downloaded at: https://www.mdpi.com/article/10.3390/jcm15155881/s1, Cohort Construction, Code Definitions, and Specifications; Figure S1: Forest plots of the subgroup analyses; Table S1: Sensitivity analyses of one-year clinical outcomes by post-TIPS NSBB strategy.

## Abbreviations

NSBB	Nonselective beta-blocker
TIPS	Transjugular intrahepatic portosystemic shunt
HE	Hepatic encephalopathy
EVB	Esophageal variceal bleeding
ICU	Intensive care unit
CSPH	Clinically significant portal hypertension
EHR	Electronic health record
HIPAA	Health Insurance Portability and Accountability Act
PSM	Propensity score matching
SMD	Standardized mean difference
HR	Hazard ratio
CI	Confidence interval
ITT	Intention to treat
BMI	Mody mass index
INR	International normalized ratio
RR	Risk ratio
RD	Risk difference
MELD-Na	Model for End-Stage Liver Disease–Sodium
FIPS	Freiburg Index of Post-TIPS Survival
KM	Kaplan–Meier
LT	Liver transplantation

## References

[1] Garcia-Tsao G., Abraldes J.G., Berzigotti A., Bosch J. (2017). Portal hypertensive bleeding in cirrhosis: Risk stratification, diagnosis, and management 2016 practice guidance by the American Association for the Study of Liver Diseases. Hepatology.

[2] Sauerbruch T., Mengel M., Dollinger M., Zipprich A., Rössle M., Panther E., Wiest R., Caca K., Hoffmeister A., Lutz H. (2015). Prevention of rebleeding from esophageal varices in patients with cirrhosis receiving small-diameter stents versus hemodynamically controlled medical therapy. Gastroenterology.

[3] Tiede A., Stockhoff L., Rieland H., Liu Z., Mauz J.B., Tergast T.L., Kabelitz M.A., Schütte S.S., Ehrenbauer A.F., Meyer B.C. (2024). No value of non-selective beta-blockers after TIPS-insertion. Aliment. Pharmacol. Ther..

[4] Kaplan D.E., Ripoll C., Thiele M., Fortune B.E., Simonetto D.A., Garcia-Tsao G., Bosch J. (2024). AASLD Practice Guidance on risk stratification and management of portal hypertension and varices in cirrhosis. Hepatology.

[5] Lee E.W., Eghtesad B., Garcia-Tsao G., Haskal Z.J., Hernandez-Gea V., Jalaeian H., Kalva S.P., Mohanty A., Thabut D., Abraldes J.G. (2024). AASLD Practice Guidance on the use of TIPS, variceal embolization, and retrograde transvenous obliteration in the management of variceal hemorrhage. Hepatology.

[6] Bureau C., Larrue H., Cortes-Cerisuleo M., Miraglia R., Procopet B., Rudler M., Trebicka J., VanWagner L.B., Hernandez-Gea V. (2025). EASL Clinical Practice Guidelines on TIPS. J. Hepatol..

[7] Billey C., Billet S., Robic M.A., Cognet T., Guillaume M., Vinel J.P., Péron J.M., Lairez O., Bureau C. (2019). A prospective study identifying predictive factors of cardiac decompensation after transjugular intrahepatic portosystemic shunt: The Toulouse algorithm. Hepatology.

[8] Ge P.S., Runyon B.A. (2016). Treatment of patients with cirrhosis. N. Engl. J. Med..

[9] Bettinger D., Sturm L., Pfaff L., Hahn F., Kloeckner R., Volkwein L., Praktiknjo M., Lv Y., Han G., Huber J.P. (2021). Refining prediction of survival after TIPS with the novel Freiburg index of post-TIPS survival. J. Hepatol..

[10] Cai W., Zheng B., Lin X., Wu W., Chen C. (2022). Prediction of patient hepatic encephalopathy risk with Freiburg index of post-TIPS survival score following transjugular intrahepatic portosystemic shunts: A retrospective study. Int. J. Gen. Med..

[11] Austin P.C. (2011). An introduction to propensity score methods for reducing the effects of confounding in observational studies. Multivar. Behav. Res..

[12] Stensrud M.J., Aalen J.M., Aalen O.O., Valberg M. (2019). Limitations of hazard ratios in clinical trials. Eur. Heart J..

[13] Young J.G., Stensrud M.J., Tchetgen Tchetgen E.J., Hernán M.A. (2020). A causal framework for classical statistical estimands in failure-time settings with competing events. Stat. Med..

[14] Schumacher M., Ohneberg K., Beyersmann J. (2016). Competing risk bias was common in a prominent medical journal. J. Clin. Epidemiol..

[15] Suissa S. (2008). Immortal time bias in pharmacoepidemiology. Am. J. Epidemiol..

[16] Miao Z., Lu J., Yan J., Lu L., Ye B., Gu M. (2020). Comparison of therapies for secondary prophylaxis of esophageal variceal bleeding in cirrhosis: A network meta-analysis of randomized controlled trials. Clin. Ther..

[17] Wijdicks E.F.M. (2016). Hepatic encephalopathy. N. Engl. J. Med..

[18] Gairing S.J., Müller L., Kloeckner R., Galle P.R., Labenz C. (2022). Review article: Post-TIPSS hepatic encephalopathy—Current knowledge and future perspectives. Aliment. Pharmacol. Ther..

[19] Friis K.H., Thomsen K.L., Laleman W., Montagnese S., Vilstrup H., Lauridsen M.M. (2023). Post-transjugular intrahepatic portosystemic shunt (TIPS) hepatic encephalopathy—A review of the past decade’s literature focusing on incidence, risk factors, and prophylaxis. J. Clin. Med..

[20] Li Y., Wu Y.T., Wu H. (2025). Management of hepatic encephalopathy following transjugular intrahepatic portosystemic shunts: Current strategies and future directions. World J. Gastroenterol..

[21] Tapper E.B., Parikh N.D. (2023). Diagnosis and management of cirrhosis and its complications: A review. JAMA.

[22] Nabilou P., Danielsen K.V., Kimer N., Hove J.D., Bendtsen F., Møller S. (2022). Blunted cardiovascular effects of beta-blockers in patients with cirrhosis: Relation to severity?. PLoS ONE.

[23] Reiberger T., Mandorfer M. (2017). Beta adrenergic blockade and decompensated cirrhosis. J. Hepatol..

[24] Téllez L., Albillos A. (2022). Non-selective beta-blockers in patients with ascites: The complex interplay among the liver, kidney and heart. Liver Int..

[25] Labenz C., Nagel M., Toenges G., Kuchen R., Schattenberg J.M., Hilscher M., Huber Y., Marquardt J.U., Labenz J., Galle P.R. (2020). Impact of non-selective β-blockers on hepatic encephalopathy in patients with liver cirrhosis. Eur. J. Intern. Med..

[26] Albillos A., Krag A. (2023). Beta-blockers in the era of precision medicine in patients with cirrhosis. J. Hepatol..

[27] Nardelli S., Riggio O., Marra F., Gioia S., Saltini D., Bellafante D., Adotti V., Guasconi T., Ridola L., Rosi M. (2024). Episodic overt hepatic encephalopathy after transjugular intrahepatic portosystemic shunt does not increase mortality in patients with cirrhosis. J. Hepatol..

[28] Xiang Y., Tie J., Wang G., Zhuge Y., Wu H., Zhu X., Xue H., Liu S., Yang L., Xu J. (2025). Post-TIPS overt hepatic encephalopathy increases long-term but not short-term mortality in cirrhotic patients with variceal bleeding: A large-scale, multicenter real-world study. Aliment. Pharmacol. Ther..

[29] Villanueva C., Albillos A., Genescà J., Garcia-Pagan J.C., Calleja J.L., Aracil C., Bañares R., Morillas R.M., Poca M., Peñas B. (2019). β blockers to prevent decompensation of cirrhosis in patients with clinically significant portal hypertension (PREDESCI): A randomised, double-blind, placebo-controlled, multicentre trial. Lancet.

[30] Ismail A., Abusuliman M., Kloub M., Abbarh S., Aloum K., Najim M.S., Al-Aquily M., Madi M.Y., Qureshi K., Syn W.-K. (2025). Comparative risk of recurrent esophageal variceal hemorrhage and other decompensation events with carvedilol versus propranolol in patients with cirrhosis: A retrospective study. Dig. Dis. Sci..

