# Supplementary material for: Continuation Versus Discontinuation of Nonselective Beta-Blockers After Transjugular Intrahepatic Portosystemic Shunt Placement: A Real-World, Target-Trial Emulation Analysis
Source: J Clin Med. 2026 Jul 28;15(15):5881. doi: 10.3390/jcm15155881 (PMC13467356; doi:10.3390/jcm15155881)
Supplement: Supplementary file 1 [file jcm-15-05881-s001.zip › jcm-4406187-supplementary.pdf]

SUPPLEMENTARY MATERIALS

Cohort Construction, Code Definitions, and Specifications

1. Data Source
Data were derived from the TriNetX U.S. Collaborative Network, a federated research platform containing de-identified electronic health record data from participating health systems. Diagnoses were identified using ICD-10-CM codes, procedures using CPT and ICD-10-PCS codes, medications using RxNorm concepts, and laboratory tests using standardized laboratory identifiers. All data were de-identified in accordance with HIPAA regulations.

2. Cohort Definitions
The study cohort comprised adults aged ≥18 years with a diagnosis of cirrhosis who underwent their first transjugular intrahepatic portosystemic shunt (TIPS; CPT 37182) and had at least one nonselective beta-blocker (NSBB) prescription recorded within the 365 days before TIPS. Patients were required to survive to post-TIPS day 90. Eligible patients were then classified during days 0–90 after TIPS as continuation (≥1 NSBB prescription) or discontinuation (no NSBB prescription) and were followed from the 90-day landmark through post-TIPS day 365.

**Domain**	**Code System**	**Codes / Definitions**
Cirrhosis	ICD-10-CM	K74.60, K74.69, K70.3
TIPS placement	CPT	37182 (first recorded procedure)
NSBB exposure	RxNorm	Propranolol; Nadolol; Carvedilol
Death (overall survival)	EHR	Structured mortality field (date of death)
Liver transplantation	CPT / ICD-10	47135; Z94.4
Hepatic encephalopathy	ICD-10-CM	K76.82
Hepatic encephalopathy-directed therapy (corroborating analysis)	RxNorm	Rifaximin; Lactulose
Esophageal variceal bleeding	ICD-10-CM	I85.01, I85.11
ICU admission	Encounter / CPT	Critical care services (CPT 99291, 99292)

3. Exposure Strategy Definitions
Continuation group: ≥1 prescription for an NSBB recorded within days 0–90 after TIPS.

Discontinuation group: no recorded NSBB prescriptions within days 0–90 after TIPS.

All patients were required to have at least one NSBB prescription within 365 days prior to TIPS placement.

4. Outcome Definitions
Primary outcomes included overall survival and transplant-free survival. Mortality was determined from the structured mortality field in the electronic record. Transplant-free survival was defined as the time from the 90-day landmark to liver transplantation or death.

Secondary outcomes included hepatic encephalopathy, esophageal variceal bleeding, and ICU admission, identified using diagnosis codes, procedure codes, and encounter constructs.

As a corroborating analysis, initiation of hepatic encephalopathy-directed pharmacotherapy (rifaximin and lactulose) was assessed as a proxy outcome, identified from RxNorm medication concepts.

5. Propensity Score Model Specification
Covariates included:

• Demographics: age, sex, race, and body mass index (kg/m^2).

• Liver disease etiology: alcoholic cirrhosis, chronic hepatitis B, chronic hepatitis C, and metabolic dysfunction-associated steatohepatitis (MASH).

• Cirrhosis- and portal hypertension-related complications: ascites, hepatic encephalopathy, esophageal variceal bleeding, spontaneous bacterial peritonitis, and hepatorenal syndrome.

• Comorbidities: hypertension, diabetes mellitus, chronic kidney disease, heart failure, and ICU admission in the year before TIPS.

• Laboratory values (nearest to the index date): hemoglobin (g/dL), platelet count ($\times 10^3/\mu L$), sodium (mEq/L), creatinine (mg/dL), albumin (g/dL), total bilirubin (mg/dL), and international normalized ratio (INR).

Nearest-neighbor 1:1 matching without replacement was performed within the platform. Covariate balance was assessed using standardized mean differences (SMDs). An SMD below 0.20 was prespecified as indicating adequate balance, and balance was additionally evaluated against the more conservative 0.10 threshold. Diagnosis- and procedure-based covariates were identified using standard ICD-10-CM and CPT concept mappings within TriNetX; laboratory covariates were the measurements nearest to the index date.

6. Design Considerations
The 90-day landmark design was selected to exclude early post-procedural instability and acute hemodynamic shifts. Patients who did not survive to day 90 were excluded to ensure comparability between exposure strategies.

Supplementary Figure S1. Forest plots of the subgroup analyses.

Hazard ratios (squares) with 95% confidence intervals (horizontal lines) for NSBB continuation versus discontinuation within each subgroup, from the 90-day landmark through 1 year. Marker area is proportional to the total number of events. Filled navy squares indicate statistically significant associations (95% CI excluding 1.0), whereas light blue squares indicate non-significant associations. n denotes the matched sample size per group. MASH, metabolic dysfunction-associated steatohepatitis; TIPS, transjugular intrahepatic portosystemic shunt.

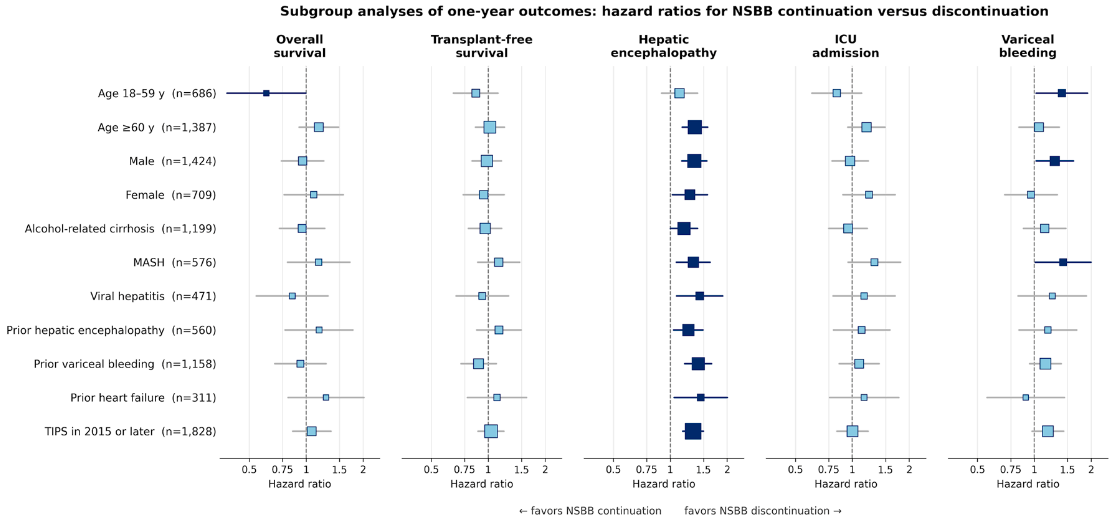

Supplementary Table S1. Sensitivity analyses of one-year clinical outcomes by post-TIPS NSBB strategy.

Analysis / outcome	Exposed, n (%)	Comparator, n (%)	KM 1-yr, %	HR (95% CI)	Log-rank p	Prop. p
Strict discontinuation (continuation vs no NSBB after TIPS, 1,859 matched pairs)						
Overall survival (death)	161 (8.7)	156 (8.4)	89.2 vs 88.0	0.89 (0.72–1.11)	0.29	0.93
Transplant-free survival	323 (17.4)	309 (16.6)	78.6 vs 76.7	0.90 (0.77–1.05)	0.18	0.54
Hepatic encephalopathy	495 (26.6)	328 (17.6)	67.3 vs 75.0	1.40 (1.22–1.61)	<0.001	0.31
Esophageal variceal bleeding	192 (10.3)	153 (8.2)	87.1 vs 88.0	1.10 (0.89–1.36)	0.38	0.27
ICU admission	221 (11.9)	171 (9.2)	85.1 vs 86.9	1.13 (0.93–1.38)	0.22	0.30
Adherence-based, per-protocol–like (sustained NSBB vs no post-TIPS NSBB, 828 matched pairs)						
Overall survival (death)	91 (11.0)	77 (9.3)	88.5 vs 86.8	0.85 (0.63–1.15)	0.29	0.36
Transplant-free survival	200 (24.2)	156 (18.8)	74.9 vs 73.9	0.94 (0.76–1.16)	0.57	0.29
Hepatic encephalopathy	352 (42.5)	167 (20.2)	56.4 vs 71.8	1.73 (1.44–2.07)	<0.001	0.74
Esophageal variceal bleeding	150 (18.1)	66 (8.0)	81.1 vs 88.5	1.71 (1.28–2.29)	<0.001	0.93
ICU admission	171 (20.7)	92 (11.1)	78.4 vs 84.2	1.39 (1.08–1.79)	0.01	0.33

Kaplan–Meier one-year probability (exposed vs comparator): survival for the survival outcomes and freedom from the event for hepatic encephalopathy, ICU admission, and esophageal variceal bleeding. The exposed group was followed longer in both analyses (adherence-based, mean 345 vs 258 days; strict-discontinuation, mean 294 vs 257 days), so crude risk ratios and risk differences are confounded by differential follow-up and are not reported; hazard ratios and Kaplan–Meier estimates account for differential follow-up. Both exposure definitions incorporate information recorded after the landmark, and the adherence-based analysis is additionally subject to immortal-time bias, so these analyses are interpreted as supportive and directional.
